# Reference Intervals for Urinary Cotinine Levels and the Influence of Sampling Time and Other Predictors on Its Excretion Among Italian Schoolchildren

**DOI:** 10.3390/ijerph15040817

**Published:** 2018-04-21

**Authors:** Carmela Protano, Roberta Andreoli, Antonio Mutti, Maurizio Manigrasso, Pasquale Avino, Matteo Vitali

**Affiliations:** 1 Department of Public Health and Infectious Diseases, Sapienza University of Rome, P.le Aldo Moro, 5, 00185 Rome, Italy; matteo.vitali@uniroma1.it; 2 Department of Medicine and Surgery, Laboratory of Industrial Toxicology, University of Parma, 43121 Parma, Italy; roberta.andreoli@unipr.it (R.A.); antonio.mutti@unipr.it (A.M.); 3 Department of Technological Innovations, National Institute for Insurance against Accidents at Work, Via IV Novembre 144, 00187 Rome, Italy; m.manigrasso@inail.it (M.M.); avino@unimol.it (P.A.); 4 Department of Agricultural, Environmental and Food Sciences (DiAAA), University of Molise, Via De Sanctis, 86100 Campobasso, Italy; 5 Institute of Ecotoxicology and Environmental Sciences, 700156 Kolkata, India

**Keywords:** cotinine, urine, children, Italy, environmental tobacco smoke exposure

## Abstract

(1) Background: Environmental Tobacco Smoke (ETS) exposure remains a public health problem worldwide. The aims are to establish urinary (u-) cotinine reference values for healthy Italian children, to evaluate the role of the sampling time and of other factors on children’s u-cotinine excretion. (2) Methods: A cross-sectional study was performed on 330 children. Information on participants was gathered by a questionnaire and u-cotinine was determined in two samples for each child, collected during the evening and the next morning. (3) Results: Reference intervals (as the 2.5th and 97.5th percentiles of the distribution) in evening and morning samples were respectively equal to 0.98–4.29 and 0.91–4.50 µg L^−1^ (ETS unexposed) and 1.39–16.34 and 1.49–20.95 µg L^−1^ (ETS exposed). No statistical differences were recovered between median values found in evening and morning samples, both in ETS unexposed and exposed. Significant predictors of u-cotinine excretions were ponderal status according to body mass index of children (β = 0.202; *p*-value = 0.041 for evening samples; β = 0.169; *p*-value = 0.039 for morning samples) and paternal educational level (*β* = −0.258; *p*-value = 0.010; for evening samples; β = −0.013; *p*-value = 0.003 for morning samples). (4) Conclusions: The results evidenced the need of further studies for assessing the role of confounding factors on ETS exposure, and the necessity of educational interventions on smokers for rising their awareness about ETS.

## 1. Introduction

Environmental Tobacco Smoke (ETS) is one of the most important environmental risk factors for human health, and a well-known threat since the late sixties, when the Surgeon General of the United States, Jesse L. Steinfeld, helped to focalize the public attention not only on the effects of smoking itself, but also on the effects of smoking on nonsmokers’ health [1]. Over the following 50 years, a growing number of adverse health effects related to ETS exposure have been demonstrated: cardiovascular diseases, respiratory disorders, lung cancer, and reproductive effects in women [1]. Besides, maternal exposure to ETS during pregnancy has been associated to several negative outcomes, such as spontaneous abortion, preterm delivery, declined birth weight, congenital malformations, facial clefts, urethral stenosis, spina bifida, diaphragmatic hernia and pigmentary anomalies, and adverse health effects in adulthood as a result of ETS exposures in utero or during childhood [2].

ETS exposure during childhood is of particular concern because children are more susceptible to toxic exposure than adults [3]. Furthermore, even if smoking bans applied in all public places in many countries have substantially reduced the ETS exposure of nonsmokers, domestic environments remain a very important source of exposure to passive smoking during childhood [4]. Nevertheless, at today, information about ETS exposure during pediatric age and potential negative effects on health is still lack compared to the need for scientific evidence. In contrast, the main international public health agencies recommend performing studies specifically devoted to the environmental children health. For this purpose, an essential tool is represented by the biomonitoring that is the measurement in biological matrices of the body burden of toxic chemical compounds, elements, or their metabolites. Biomonitoring studies consent to trace exposure profiles and let to elaborate specific reference values, useful to perform comparisons and risk evaluation processes. A very recent research was performed on adults, with the aim to examine the behavioral and sociodemographic factors affecting the levels of urinary (u-) cotinine in ETS exposed individuals, and to derive reference range for u-cotinine in adult population. The results evidenced that significant predictors of u-cotinine concentration were living with smokers, being exposed to smoke in domestic environment, the duration of ETS exposure, and the time between the last exposure and urine collection; furthermore, an upper reference value to identify environmental exposure to ETS was proposed [5]. However, these new findings cannot be used for children’s evaluation. In our previous researches we demonstrated, by the use of some biological indicators of ETS exposure such as u-benzene [6] and u-cotinine [7], that ETS exposure of children is strongly related to parental smoking and their habits at home. In the same study population, we recovered a significant association between u-benzene and u-cotinine with specific biomarkers for oxidative damage to DNA, demonstrating the potential health threats derived from ETS exposure in pediatric age. Nevertheless, we did not evaluate the contribution of the sampling time on u-cotinine excretion because we quantified cotinine concentrations only in a spot urine sample collected, for each child, at the end of the day. Furthermore, we did not elaborate specific reference intervals for u-cotinine concentrations in ETS exposed and not exposed children. Another recent study, carried out by using data from the DEMOCOPHES study population for Romania, Portugal and Poland, elaborated reference intervals for u-cotinine in children exposed to ETS. However, the same study demonstrated a significant difference in u-cotinine distribution of children living in different countries, suggesting the necessity of country-specific researches in order to trace appropriate exposure profiles [8].

The aims of the present study were: (1) to establish the reference values of u-cotinine among a large group of healthy Italian children grouped according to ETS exposure status; (2) to evaluate the role of the collection time and of other potential interfering/confounding factors (such as gender, age, body mass index, BMI, parental educational level) on u-cotinine excretion during paediatric age.

## 2. Materials and Methods

### 2.1. Study Population and Design

A cross-sectional human biomonitoring survey was performed on a sample of healthy schoolchildren, aged between 5 and 11 years, living in Central Italy and attending some primary school districts. Details on the selected areas, the enrolment of children, and methods for gathering information on participants and for collecting urine samples were previously reported [9,10,11,12,13,14,15]. Briefly, information on children and their parents (gender, birth date, height, weight, exposure to ETS, parents’ educational level, and the activities taken place during the sampling day) was collected through an ad hoc questionnaire filled in by parents together with the informed consent.

The research protocol, together with the questionnaire and the informed consent forms, were approved by the Ethical Committee of the teaching hospital Policlinico Umberto I of Rome, Italy (Protocol n. 2894/12.09.2013).

### 2.2. Sample Collection and Analytical Determinations

Parents of each participant collected two spot urine samples of the child, the first in the evening of a previously agreed weekday (the last emiction before going to sleep) and the second in the early morning of the day after (the first emiction after waking up). Each sample was collected in a metal-free polyethylene high-density bottle and immediately refrigerated and kept at 4 °C until the delivery to the laboratory, where each sample was subdivided in aliquots and frozen at −20 °C until the analyses. The analytical method used for detecting u-cotinine and u-creatinine is described in detail in previous papers [7,16] and is applied with minor modifications. For u-cotinine determination urine samples were added with the internal standard (cotinine-d^3^), centrifuged at 10,000× *g* for 10 min and analyzed by isotopic dilution liquid chromatography tandem mass spectrometry (LC–MS/MS). Chromatography was performed on an Atlantis^®^ dC_18_ column (100 × 2.0-mm i.d., 3 µm; Waters, Milford, MA, USA) using variable proportions of 10 mM aqueous formic acid (pH 3.75) and methanol. Analytes were ionized in positive-ion mode and the transitions chosen for selected reaction monitoring detection of cotinine and its internal standard were *m/z* 177 → 80 and *m*/*z* 180 → 101, respectively. The limit of detection was 0.2 µg L^−1^ (5 µL injected), the coefficient of variation of the method (expressed as CV%) was below 2% for all intra- and inter-day determinations, calculated at three different spiked levels (2, 10 and 20 µg L^−1^, respectively). Urinary creatinine was measured by the method of Jaffe [17].

### 2.3. Covariates Gathered by the Questionnaires

Gender was categorized as 0 = male and 1 = female, while age was defined as a continuous variable, calculated as the difference between the date of the sampling day and the birth date.

ETS exposure status was based on the cohabitants’ smoking habits: if the child lived with at least one smoker, he/she was considered to be exposed to ETS; children were consequently grouped as 0 = unexposed and 1 = exposed.

The BMI of each participant was calculated according to the weight and height reported on the questionnaire by parents. The BMI values were used to categorize the children in four groups—underweight, normal weight, overweight or obese—according to sex—specific BMI—for—age growth charts produced by the International Obesity Task Force growth curves [18]. Then, each child was classified according to his/her ponderal status according to BMI as follows:

- 1 = thinness of 1st, 2nd or 3rd degree;
- 2 = normal weight;
- 3 = overweight;
- 4 = obesity.

For univariate and multivariate analyses, children were categorized as follows: 0 = thinness of 1st, 2nd or 3rd degree and normal weight, and 1 = overweight and obesity.

Educational level of each parent was coded according to the Organisation for Economic Cooperation and Development (OECD) as follows: 1 = Basic (≤9 years); 2 = Upper secondary (≤14 years); 3 = Tertiary/higher (≥17 years). For univariate and multivariate analyses, educational level was re-categorized as 0 = until to upper secondary and 1 = tertiary/higher.

### 2.4. Statistical Analysis

Statistical elaboration was performed using the statistical softwares SPSS 22 (IBM Corp., Armonk, NY, USA) and 15.2 MedCalc (MedCalc Software, Mariakerke, Belgium).

The research project was presented to 619 children and the participation rate was 70%, for a total of 434 participants. However, statistical analyses were carried out on data related to 330 children, for a total of 660 urine samples, while data on 104 participants were excluded for one of the following reasons: (1) urine sample was not sufficient for determining cotinine and/or creatinine concentrations; (2) urine sample was too diluted or too concentrated (level of creatinine <0.3 g L^−1^ or >3.0 g L^−1^, respectively); (3) participant had returned just one of the two urine samples; (4) participant had at least one parent who was not Italian. The last reason was in order to elaborate reference values for Italian children and to avoid the influence of the ethnicity, a known factor influencing metabolism and excretion of substances from the body [19].

First of all, the normality of the distribution of u-cotinine concentration, separately for evening and morning urine samples and for exposure to ETS, was assessed using the one-sample Kolmogorov-Smirnov test. In all cases, the u-cotinine levels were not normally distributed. Consequently, the elaboration of the reference ranges was carried out by non-parametric methods and the reference limits were estimated as the 2.5th and 97.5th percentiles of the distribution, as recommended by the guidelines of the National Committee for Clinical Laboratory Standards (NCCLS) and the Clinical and Laboratory Standards Institute (CLSI). The presence of outliers was evaluated by the use of Reed’s one-third rule. Besides, median values of u-cotinine concentrations found respectively in evening and morning samples of children unexposed and exposed to ETS, were compared by the use of the Mann-Whitney test. The same test was also used to compare median levels of u-cotinine according to gender. Furthermore, correlation among u-cotinine, u-creatinine, and age was tested by the use of the Spearman’s rank correlation coefficients, independently for both evening and morning samples.

Further statistical analyses were carried out to evaluate the influence on u-cotinine excretion of some selected interfering or confounding variables. In particular, after the log-transformation of data (ln), univariate and multivariate analyses were performed in order to assess the contribute of ponderal status according to BMI and the educational level of both father and mother on u-creatinine concentration. Thus, the Student’s t-test was used to compare u-cotinine levels based on ponderal status according to BMI and on the educational level of parents. Then, we proceeded to run four multiple linear regression analyses: the first two models (one for evening urine samples and one for morning urine samples) were run considering the u-cotinine concentration of all children as the dependent variable and gender, age, creatinine, ETS exposure, ponderal status according to BMI, and educational level of the mother and father as independent variables. Besides, additional two models (one for evening urine samples and one for morning urine samples) were run with the same dependent and independent variables (with the exception of ETS exposure status), but considering only children who were considered exposed to ETS. Forward linear regression analyses were performed using a significance level of 0.05 for entry and 0.10 for removal from the model. The significance level for all analyses was *p* ≤ 0.05 (two tailed). The “goodness of fit” of the model was assessed using R^2^ statistics.

## 3. Results

### 3.1. Characteristics of the Study Population

Table 1 shows the characteristics of the study population. The sample was composed of 54.7% of males and 45.3% of females and presented a age arithmetic mean of 8.6 years old. Information obtained from the questionnaires evidenced that more than one third of the studied children were exposed to ETS and, according to the BMI, more than one quarter of them were overweight or obese. As regard the educational levels of parents, 41% of mothers and 38% of fathers were graduated. Mean u-creatinine levels in evening samples were equal to 1.00 (± 0.69) and 1.11 (± 0.39) g L^−1^, respectively.

### 3.2. Reference Values of u-Cotinine Levels

Reference values of u-cotinine concentrations, calculated separately for evening and morning samples for children not exposed and exposed to ETS, considering all participants and according to gender, are presented in Table 2 and Table 3 (expressed as µg L^−1^ and µg g^−1^ creatinine, respectively).

**Figure 1:**
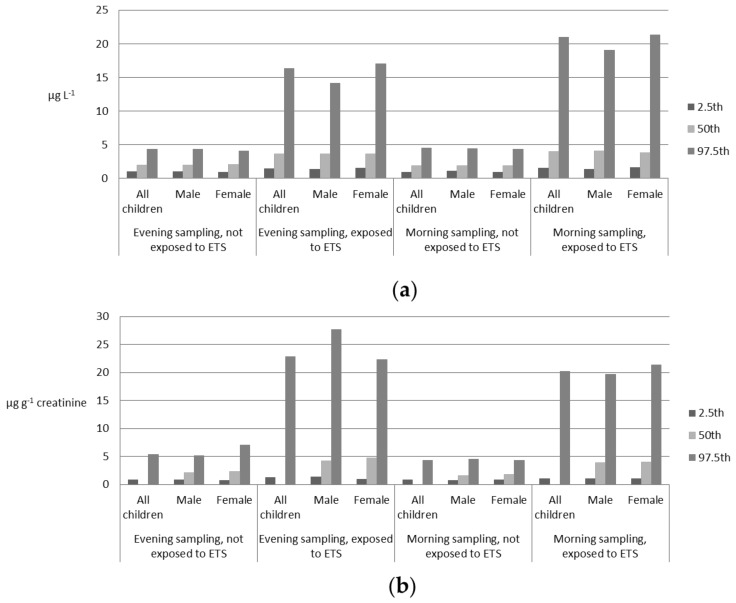
Reference values of urinary cotinine levels (expressed both as µg L^−1^ and µg g^−1^ creatinine) for children not exposed and exposed to Environmental Tobacco Smoke (ETS) calculated for all participants and according to gender: (**a**) urinary cotinine levels expressed both as µg L^−1^; (**b**) urinary cotinine levels expressed both as µg g^−1^ creatinine.

The reference ranges (both expressed as µg L and µg g^−1^ creatinine) are reported in Figure 1. The reference intervals obtained in the evening samples were respectively equal to 0.98–4.29 µg L^−1^ for children not exposed to ETS and 1.39–16.34 µg L^−1^ for children exposed to ETS. Similarity, reference intervals elaborated in morning samples were respectively equal to 0.91–4.50 µg L^−1^ for children not exposed to ETS and 1.49–20.95 µg L^−1^ for children exposed to ETS. Statistical significant differences were found comparing median values of children unexposed and exposed to ETS, considering separately evening and morning samples. On the other hand, no differences were recovered comparing median values of evening and morning samples, considering separately the ETS unexposed group and the ETS exposed group. Besides, no differences were found comparing u-cotinine median levels of males and females. Similar results were found using values expressed as µg g^−1^ creatinine, with one exception: median values of evening and morning samples were significantly different; probably this difference is related to the difference between u-creatinine values and not between u-cotinine.

Table 4 shows the Spearman’s rank correlation coefficients among u-cotinine levels, u-creatinine and age, separately for evening and morning samples and for exposure to ETS. The results evidence a significant positive relationship between u-cotinine and u-creatinine levels, except for the subgroup of morning samples of children exposed to ETS. No significant correlation was found between the concentrations of u-cotinine or u-creatinine and age.

### 3.3. The Contribution of the Covariates on u-Cotinine Levels

In Table 5 and Table 6 are reported the results of univariate analyses carried out to evaluate the relationship between the concentration of u-cotinine (independently for evening and morning urine samples) and the ponderal status according to the BMI, the mother’s and father’s educational level.

As showed in Table 5, overweight and obese children had u-cotinine levels significantly higher than thin and normal weight children. Besides, u-cotinine levels were significant higher in children having at least one parent with a low educational level. Univariate analysis performed using values expressed as µg g^−1^ creatinine showed similar differences with the exception of the comparison of median values recovered respectively for thinness of 1st, 2nd or 3rd degree and normal weight group and for overweight and obesity group. This result can be attributed to differences in u-creatinine and not in u-cotinine itself. The results of univariate analyses were partially confirmed by multivariate analysis (Table 7 and Table 8).

Considering the whole sample of children, significant predictors of u-cotinine excretion for both evening and morning urine samples were exposure to ETS (β-coefficient = 0.594; *p*-value < 0.001 for evening samples; β-coefficient = 0.641; *p*-value < 0.001 for morning samples) and u-creatinine levels (β-coefficient = 0.207; *p*-value < 0.001 for evening samples; β-coefficient = 0.158; *p*-value = 0.001 for morning samples). These regression models explained 38.0% and 45.1% of the variances of u-cotinine in evening and morning samples respectively. Considering only the group of children exposed to ETS, two results of the univariate analyses were confirmed for both evening and morning urine samples: ponderal status according to BMI and father’s educational level displayed a significant negative independent role on u-cotinine excretion.

## 4. Discussion

Despite the well-known adverse effects related to ETS exposure, especially when it occurs early during life, and the prevention strategies implemented for reducing this exposure, at today, this is still a relevant problem for public health worldwide. Indeed, this is demonstrated also by the percentage of children exposed to ETS found in our study: more than one third of the participants resulted exposed to ETS in domestic environment. Consequently, it is essential to trace exposure profiles and to investigate the variables influencing the exposure. In this context, u-cotinine is a useful biological indicator, but there are some critical points that prompted our study. First of all, at today there is a lack of reference ranges of u-cotinine concentrations specifically elaborated for Italian children that, instead, represent the baseline for performing comparison and for evaluating the exposure during paediatric age. Secondly, it is essential to understand the best time window of the day in which cotinine is excreted at higher amount and, thus, the best moment of the day in which collecting urine sample. In particular, two moments of the day are typically considered suitable for collecting urine samples: the last emiction of the evening and the first emiction of the morning, when individuals are at home, and they can serenely collect the sample, immediately refrigerate and maintain it at 4 °C. Finally, it is essential to investigate the influence of confounding and interfering variables on u-cotinine excretion, in order to fully understand the exposure profile during paediatric age.

In our knowledge, this is the first study that elaborates u-cotinine level reference ranges for Italian children. First of all, it is important to note the high variability of the reference ranges among children exposed to ETS, both considering evening and morning samples. The wide range for u-cotinine concentration is in part related to some interfering/confounding factors that we did not consider, and in gran part can be attributed to the smoking habits of cohabitants smokers at home, such as complete, partial or no smoking ban in domestic environment, the number of smokers, and the number of cigarettes smoked by each smoker at home.

Median values reported here are very similar to those found in our previous study performed in a sample of children aged 5–11 years old and living in the same monitored areas in the years 2007–2009 (median values of u-cotinine in the evening samples equal to 1.79 µg L^−1^ and 3.90 µg L^−1^ in children unexposed and exposed to ETS, respectively) [7]. Moreover, similar results were obtained in another our study performed on children aged 5–11 years and living near and far away an oil refinery in Sicily Region (Southern Italy). In that study, we observed a decrease in u-cotinine levels in the urines of the morning sampling (geometric mean 1.20 [1.60] μg g^−1^ creatinine) compared to the evening one (μg g^−1^ creatinine) but only in the restricted group of children living near the oil refinery and unexposed to ETS [20]. The comparison of our results with those reported from other European countries evidences median values of the same order of magnitude, even if the concentrations recovered in the present study for children ETS exposed are lower than those found in the other countries [8]. The cited study, performed on children living in Romania, Portugal and Poland, reported whole median values for u-cotinine equal to 0.8 and 6.1 µg L^−1^ for children unexposed and exposed to ETS, respectively. However, considering the single countries, significant differences between the u-cotinine concentrations were recovered for children exposed to ETS, with significant higher levels in Romania and significant lower levels in Portugal. These results reflect differences in smoking prevalence of studied countries, and demonstrate the necessity of country-specific evaluation and references ranges elaboration. Moreover, the comparison of u-cotinine levels found in the present study with the data reported in the scientific literature on Italian adults confirm that there are differences, even if not relevant, in the excretion of u-cotinine between pediatric and adult populations. Indeed, a recent study performed on Italian adults [5] found medians values of u-cotinine respectively equal to 0.39 and 1.38 µg L^−1^ for unexposed and exposed to ETS, that are two-three times lower than our median values, both for the two groups. Given the differences recovered not only between exposed to ETS, but also in the unexposed ones, this difference can be attributed to the different capacity to metabolize drug between children and adults, that involve differences in the percentage and rate of nicotine transformed into cotinine. Another explanation could be related to the higher elimination rate from plasma in childhood respect to the adults, well known for many other substances [21].

As regard to the influence of the sampling time on u-cotinine excretion, we did not find statistically significant differences between median values obtained from evening and morning samples, both for unexposed or exposed groups. This result suggests that collecting the urine of the first or the last emiction of the day does not influence the u-cotinine excretion and, thus, sampling could be performed indifferently in these two moments. Our finding differs from the results of a previous report [5], that evidenced statistically significant differences in u-cotinine levels according to the time from last exposure. The authors found median values equal to 3.55, 1.76, and 0.75 µg L^−1^, respectively, for urine samples collected <5 h, 5–24 h, >24 h from the last exposure. However, an interval of 19 h (from 5 to 24 h after the last exposure) is probably too long considering the average half-life of 16 h of cotinine [22]; it should be more appropriate to identify two or more sampling times within this interval.

Additional relevant findings are related to the predictors that influence the u-cotinine excretion. First of all, we found that overweight and obese children excreted a major amount of u-cotinine respect to those with a normal weight or underweight. This result, as evidenced by the multivariate analysis, affects only children exposed to ETS. Thus, it is strongly related to ETS exposure. It is well-known that several confounding factors such as educational levels, socio-economic status, and lifestyle habits are worse in smoking than in non-smoking parents, but we found an independent role of ponderal status according to BMI of children respect to the educational levels of both father and mothers. Some previous reports demonstrated a significant association between the maternal smoking or ETS exposure during pregnancy and the increase of child BMI, suggesting that ETS exposure may contribute to the pathogenesis of obesogenic child growth [23,24]. One explanation to this association is related to the ability of exposure to ETS to active hormonal systems, which influence metabolic programming and/or to increase child appetite and circulating leptin levels [24]. Besides, another recent study on adults evidenced that exposure to ETS was associated not only with obesity, but also worsening glycemic parameters, suggesting the potential risk of diabetes as another adverse effect resulting from ETS exposure [25].

Another significant inverse relationship was recovered between education level of father and u-cotinine levels in children exposed to ETS, according to the results of previous studies [26,27]. Indeed, another recent study [28] focalized the attention on the contribution of fathers’ smoking behavior and ETS exposure of children at home. The results of the cited study evidenced that, as expected, u-cotinine concentrations of children exposed to ETS at home were positively associated with the number of cigarettes smoked in front of the children at home per day, the number of cigarettes smoked by the father in front of the children at home, and the mean duration of the children’s exposure to ETS at home. Besides, 97.6% of the smoking adults cohabiting with the study children did not follow any smoking restrictions at home. All these data revealed that a great percentage of parents, especially the fathers, denied the ETS exposure of their children at home. This is of particular concern considering the recent evidences about the second- and third-hand smoke in indoor environment, that involve a lack of protection of children from ETS exposure [29]. Consequently, programs aimed to make homes smoke-free should be targeted at all family members, as already strongly recommended previously [30]. However, while many previous reports [27] evidenced the independent role of maternal educational level on ETS exposure at home, we find this association only in the univariate analysis, but not in the multivariate one. This result confirms the presence of multiple factors influencing ETS exposure and complex association between these variables, highlighting the necessity to study in depth the interfering/confounding variables of ETS exposure.

This study has some limitations. First of all, it is a cross-sectional study; thus, the predictors and the effects were evaluated at one moment in time. Secondly, we consider children exposed to ETS if they lived with at least one smoker; thus we excluded the possible contribution of exposure outside home. However we investigated, through the questionnaire, all the activities carried out by the participants during the monitoring day, and we did not find situations “at risk” of ETS exposure. Moreover, we did not investigate other relevant confounding factors such as socio-economic status. It should be interesting to evaluate also this factor in order to examine if it could influence ETS exposure or the other interfering variables, such as ponderal status according to BMI of children or educational levels of fathers. Finally, apart from u-cotinine and u-creatinine that were quantified in laboratory, the other information used for the statistical elaboration was taken from the questionnaire. This choice makes information less precise, but allows us to study a great number of individuals.

## 5. Conclusions

This is the first study that elaborated reference u-cotinine range values for Italian children, useful to trace exposure profile and to evaluate u-cotinine distribution and ETS exposure status. The other main findings of the present study are related to some potential interfering/confounding factors on u-cotinine excretion. First of all, we found that u-cotinine levels were similar in urine samples collected in the evening or in the morning after; demonstrating that samples can be collected indifferently during the last emiction of the night or the first one of the next morning. Besides, we found that ponderal status according to BMI of children was a significant independent predictor of u-cotinine levels; this result highlights another potential threat of ETS exposure on human health. Finally, a lower paternal education increase u-cotinine levels, evidencing the need of educational interventions on smokers, in order to raise their awareness about ETS exposure and the need of smoke-free home for protecting the health of non-smokers cohabitants.

## Figures and Tables

**Table 1:** Characteristics of the study population.

Variable		Descriptives in % If Not Stated Otherwise (N)
Age		Mean 8.60 (standard deviation 1.38)
Gender	Male	54.7 (N = 180)
	Female	45.3 (149)
Exposure to environmental tobacco smoke	Not exposed	63.6 (N = 208)
	exposed	36.4 (N = 119)
Ponderal status according to body mass index	Thinness of 1st, 2nd or 3rd degree	9.2 (N = 26)
	Normal weight	67.7 (N = 191)
	Overweight	18.4 (N = 52)
	Obesity	4.6 (N = 13)
Maternal education (years)	Basic (≤9 years)	14.1 (N = 46)
	Upper secondary (≤14 years)	44.8 (N = 146)
	Tertiary/higher (≥17 years)	41.1 (N = 134)
Paternal education (years)	Basic (≤9 years)	20.7 (N = 66)
	Upper secondary (≤14 years)	41.4 (N = 132)
	Tertiary/higher (≥17 years)	37.9 (N = 121)

**Table 2:** Reference values of urinary cotinine levels (expressed as µg L^−1^) for children not exposed and exposed to Environmental Tobacco Smoke (ETS) calculated for all participants and according to gender.

Variable		2.5th	50th	97.5th
Evening sampling Not exposed to ETS	All children	0.98	1.97 ^1,7^	4.29
	Male	0.98	1.92 ^2^	4.36
	Female	0.93	2.03 ^2^	4.04
Evening sampling Exposed to ETS	All children	1.39	3.66 ^1,8^	16.34
	Male	1.34	3.63 ^3^	14.14
	Female	1.52	3.66 ^3^	17.03
Morning sampling Not exposed to ETS	All children	0.91	1.83 ^4,7^	4.50
	Male	1.04	1.83 ^5^	4.39
	Female	0.86	1.83 ^5^	4.36
Morning sampling Exposed to ETS	All children	1.49	3.97 ^4,8^	20.95
	Male	1.35	4.05 ^6^	19.07
	Female	1.61	3.78 ^6^	21.36

^1^ *p*-Value < 0.001, Mann-Whitney test (all children evening sampling, not exposed vs. exposed to ETS). ^2^ *p*-Value = 0.611, Mann-Whitney test (evening sampling not exposed to ETS, male vs. female). ^3^ *p*-Value = 0.983, Mann-Whitney test (evening sampling exposed to ETS, male vs. female). ^4^ *p*-Value < 0.001, Mann-Whitney test (all children morning sampling, not exposed vs. exposed to ETS). ^5^ *p*-Value = 0.333, Mann-Whitney test (morning sampling not exposed to ETS, male vs. female). ^6^ *p*-Value = 0.749, Mann-Whitney test (morning sampling exposed to ETS, male vs. female). ^7^ *p*-Value = 0.332, Mann-Whitney test (all children not exposed to ETS, evening vs. morning sampling). ^8^ *p*-Value = 0.342, Mann-Whitney test (all children exposed to ETS, evening vs. morning sampling).

**Table 3:** Reference values of urinary cotinine levels (expressed as µg g^−1^ creatinine) for children not exposed and exposed to Environmental Tobacco Smoke (ETS) calculated for all participants and according to gender.

Variable		2.5th	50th	97.5th
Evening sampling Not exposed to ETS	All children	0.85	2.20 ^1,7^	5.41
	Male	0.89	2.09 ^2^	5.18
	Female	0.79	2.32 ^2^	7.08
Evening sampling Exposed to ETS	All children	1.33	4.35 ^1,8^	22.88
	Male	1.38	4.27 ^3^	27.75
	Female	0.98	4.74 ^3^	22.37
Morning sampling Not exposed to ETS	All children	0.84	1.75 ^4,7^	4.39
	Male	0.80	1.65 ^5^	4.55
	Female	0.84	1.83 ^5^	4.39
Morning sampling Exposed to ETS	All children	1.07	3.93 ^4,8^	20.18
	Male	1.05	3.89 ^6^	19.67
	Female	1.06	4.05 ^6^	21.37

^1^ *p*-Value < 0.001, Mann-Whitney test (all children evening sampling, not exposed vs. exposed to ETS). ^2^ *p*-Value = 0.200, Mann-Whitney test (evening sampling not exposed to ETS, male vs. female). ^3^ *p*-Value = 0.556, Mann-Whitney test (evening sampling exposed to ETS, male vs. female). ^4^ *p*-Value< 0.001, Mann-Whitney test (all children morning sampling, not exposed vs. exposed to ETS). ^5^ *p*-Value = 0.198, Mann-Whitney test (morning sampling not exposed to ETS, male vs. female). ^6^ *p*-Value = 0.950, Mann-Whitney test (morning sampling exposed to ETS, male vs. female). ^7^ *p*-Value < 0.001, Mann-Whitney test (all children not exposed to ETS, evening vs. morning sampling). ^8^ *p*-Value = 0.152, Mann-Whitney test (all children exposed to ETS, evening vs. morning sampling).

**Table 4:** Spearman’s rho among u-cotinine levels (µg L^−1^), u-creatinine (g L^−1^) and age separately for evening and morning samples and for ETS exposure.

Variable		u-Creatinine Spearman’s rho (*p*-Value)	Age Spearman’s rho (*p*-Value)
Evening samples Not exposed to ETS	u-cotinine	0.291 (<0.000)	−0.004 (0.959)
	u-creatinine		0.131 (0.059)
Morning samples Not exposed to ETS	u-cotinine	0.301 (0.001)	−0.057 (0.418)
	u-creatinine		0.125 (0.073)
Evening samples Exposed to ETS	u-cotinine	0.199 (0.030)	0.086 (0.363)
	u-creatinine		0.172 (0.066)
Morning samples Exposed to ETS	u-cotinine	0.084 (0.362)	0.121 (0.196)
	u-creatinine		0.172 (0.066)

**Table 5:** Univariate analyses of the u-cotinine levels expressed as µg L^−1^ (independently for evening and morning urine samples) for ponderal status according to Body Mass Index (BMI), mother's and father’s educational level.

Variable		Evening Samples Median [IQR] GM [GSD] ^1^	*p*-Value	Morning Samples Median [IQR] GM [GSD]	*p*-Value
Ponderal status according to BMI	Thinness of 1st, 2nd or 3rd degree and normal weight	2.17 [1.52] 2.35 [0.53]	0.001	2.00 [0.58] 2.34 [0.59]	0.009
	Overweight and obesity	2.73 [2.23] 3.09 [0.68]		2.77 [2.93] 2.96 [0.74]	
Maternal education (years)	Until to upper secondary (≤14 years)	2.46 [2.39] 2.89 [0.65]	<0.001	2.40 [2.91] 2.92 [0.73]	<0.001
	Tertiary/higher (≥17 years)	2.07 [2.09] 2.14 [0.47]		1.93 [0.81] 2.06 [0.49]	
Paternal education (years)	Until to upper secondary (≤14 years)	2.44 [2.44] 2.88 [0.67]	<0.001	2.35 [2.82] 2.88 [0.75]	<0.001
	Tertiary/higher (≥17 years)	2.09 [1.04] 2.10 [0.41]		1.95 [0.81] 2.03 [0.41]	

^1^ GM = Geometric Mean; GSD = Geometric Standard Deviation.

**Table 6:** Univariate analyses of the u-cotinine levels expressed as µg g^−1^ creatinine (independently for evening and morning urine samples) for ponderal status according to Body Mass Index (BMI), mother's and father’s educational level.

Variable		Evening Samples Median [IQR] GM [GSD] ^1^	*p*-Value	Morning Samples Median [IQR] GM [GSD]	*p*-Value
Ponderal status according to BMI	Thinness of 1st, 2nd or 3rd degree and normal weight	2.55 [2.05] 2.71 [0.61]	0.131	2.02 [1.71] 2.24 [0.62]	0.004
	Overweight and obesity	2.86 [3.02] 3.17 [0.77]		2.83 [2.76] 2.96 [0.73]	
Maternal education (years)	Until to upper secondary (≤14 years)	2.97 [3.06] 3.16 [0.75]	0.002	2.47 [2.86] 2.77 [0.73]	0.001
	Tertiary/higher (≥17 years)	2.43 [1.60] 2.48 [0.53]		1.88 [1.44] 2.06 [0.52]	
Paternal education (years)	Until to upper secondary (≤14 years)	2.91 [3.07] 3.17 [0.74]	0.002	2.41 [2.70] 2.74 [0.74]	0.001
	Tertiary/higher (≥17 years)	2.41 [1.51] 2.39 [0.51]		1.88 [1.46] 1.969 [0.45]	

^1^ GM = Geometric Mean; GSD = Geometric Standard Deviation.

**Table 7:** Significant predictors of the urinary (u-) concentration of cotinine expressed as µg L^−1^ (independently for evening and morning urine samples).

	Independent Variable	B (Regression Coefficient)	Standard Error	(Regression Standardized Coefficient)	*p*-Value	Adjusted R^2^
**Evening samples**	Constant	0.358	0.078	-	<0.001	0.380
	Exposure to ETS	0.726	0.059	0.594	<0.001	
	u-creatinine	0.304	0.071	0.207	<0.001	
**Morning samples**	Constant	0.311	0.089	-	0.001	0.451
	Exposure to ETS	0.852	0.061	0.641	<0.001	
	u-creatinine	0.259	0.075	0.158	0.001	

Variables included in the multiple linear regression models (forward method): gender (female vs. male), age (as continuous variable), urinary creatinine (u-creatinine as a continuous variable), exposure to ETS (ETS-exposed vs. ETS-unexposed), ponderal status according to the BMI (thinness of 1st, 2nd or 3rd degree/normal weight vs. overweight/obesity), mother's educational level, and father's educational level.

**Table 8:** Significant predictors of the urinary (u-) concentration of cotinine expressed as µg L^−1^ (independently for evening and morning urine samples) in children exposed to ETS.

	Independent Variable	B (Regression Coefficient)	Standard Error	(Regression Standardized Coefficient)	*p*-Value	Adjusted R^2^
**Evening samples**	Constant	0.954	0.168	-	<0.001	0.131
	Ponderal status according to BMI	0.272	0.131	0.202	0.041	
	Paternal education (years)	−0.423	0.162	−0.258	0.010	
	u-creatinine	0.429	0.169	0.253	0.013	
**Morning samples**	Constant	1.112	0.209	-	<0.001	0.089
	Ponderal status according to BMI	0.239	0.143	0.169	0.039	
	Paternal education (years)	−0.536	0.176	−0.013	0.003	
	u-creatinine	0.386	0.177	0.224	0.031	

Variables included in the multiple linear regression models (forward method): gender (female vs. male), age (as continuous variable), urinary creatinine (u-creatinine as a continuous variable), ponderal status according to the body mass index (thinness of 1st, 2nd or 3rd degree/normal weight vs. overweight/ obesity), mother's educational level, and father’s educational level.
